# Characterization and phylogenetic analysis of the complete mitochondrial genome of the human pathogenic fungus *Cryptococcus* sp. (Tremellales: Cryptococcaceae)

**DOI:** 10.1080/23802359.2019.1693295

**Published:** 2019-11-21

**Authors:** Yue Chen, Xiaorong Sun, Wenjuan Gui, Qianru Zhang, Dan Su

**Affiliations:** a Chengdu Bio-HT Company Limited, Chengdu, China;; b General Hospital of Ningxia Medical University, Yinchuan, China;; c West China School of Public Health and West China Fourth Hospital, Healthy Food Evaluation Research Center, Sichuan University, Chengdu, China;; d State Key Laboratory of Biotherapy and Cancer Center, West China Hospital, Sichuan University, Chengdu, China;; e Guangdong Raynovent Biotech Co., Ltd., Guangdong, China;; f Guangdong Zhongsheng Pharmaceutical Co., Ltd., Guangdong, China

**Keywords:** *Cryptococcus*, mitochondrial genome, phylogenetic analysis

## Abstract

In the present study, the complete mitogenome of *Cryptococcus* sp. were sequenced and assembled. The complete mitogenome of *Cryptococcus* sp. was composed of circular DNA molecules, with a total length of 30,029 bp. The base composition of this mitochondrial genome is as follows: A (31.94%), T (34.89%), G (15.97%), and C (17.21%). The mitogenome contains 20 protein-coding genes, 2 ribosomal RNA genes (rRNA), and 22 transfer RNA (tRNA) genes. Phylogenetic analysis showed that the mitogenome of the *Cryptococcus* sp. exhibited a closest relationship with *Cryptococcus gattii*.

Cryptococcosis is a worldwide invasive fungal infection caused by *Cryptococcus* species (May et al. 2016). Cryptococcosis affects the central nervous system, lung, skin, and other body parts of human and other mammals, Endangers human life and brings great challenges to treatment. *Cryptococcus neoformans* and *C. gattii* complex are the pathogens of cryptococcosis, which are the main factors leading to the death of individuals with low immune function. According to the structural variations, molecular characteristics, and genetic sequence, *C. neoformans* and *C. gattii* complex can be subdivided into varieties, serotypes, and molecular types (Cuomo et al. 2018). It is known that in the natural and clinical environment, the haploid lineages within and between the two species complexes resulted in intraspecific and interspecific diploid/aneuploid hybrid strains (Samarasinghe and Xu 2018). Since their initial discovery in 1977 (Bennett et al. 1977), cryptococcal hybrids have been found more and more in clinical and environmental settings. More than 30% of cryptococcal infections in some parts of Europe are caused by hybrid strains. The mitogenome of *Cryptococcus* sp. reported here will promote further understanding of the population genetics, evolution and pathogenicity of this fungal complex.

The specimen (*Cryptococcus* sp.) was isolated from the seedling of a conifer in Chengdu, Sichuan, China (104.52 E; 34.16 N) and was stored in Chengdu Bio-HT Company Limited (No. MNC1). This strain was identified as belonging to the *C. gattii* complex. The Fungal DNA Kit D3390-00 (Omega Bio-Tek, Norcross, GA, USA) was used to extract the total genomic DNA of *Cryptococcus* sp. The total genomic DNA was purified through a Gel Extraction Kit (Omega Bio-Tek, Norcross, GA, USA). Purified genomic DNA was stored in the sequencing company (BGI Tech, Shenzhen, China). We constructed sequencing libraries with purified DNA following the instructions of NEBNext^®^ Ultra™ II DNA Library Prep Kit (NEB, Beijing, China). Whole genomic sequencing was performed by the Illumina HiSeq 2500 Platform (Illumina, SanDiego, CA, USA) (Chen et al. 2019). Multiple steps were used for quality control and assembly of the mitogenome (Li, Liao et al. 2018; Li, Wang et al. 2018). Briefly, the *Cryptococcus* sp. mitogenome was *de novo* assembled using the SPAdes 3.9.0 software (Bankevich et al. 2012). MITObim V1.9 (Hahn et al. 2013) was used to fill gaps among contigs. The complete mitogenome was annotated using the MFannot tool (Valach et al. 2014), combined with manual corrections. tRNA genes were predicted using tRNAscan-SE v1.3.1 (Lowe and Chan 2016).

The total length of the *Cryptococcus* sp. mitogenome is 30,029 bp. This mitogenome was submitted to GenBank database under Accession No. MN623378. The circular mitogenome contains 20 protein-coding genes, 2 ribosomal RNA genes (rRNA), and 22 transfer RNA (tRNA) genes. The base composition of this mitochondrial genome is as follows: A (31.94%), T (34.89%), G (15.97%), and C (17.21%).

To validate the phylogenetic position of *Cryptococcus* sp., we construct a phylogenetic tree of 14 closely related species based on the nucleotide sequences of the 14 core PCGs (*atp6, atp8, atp9, cob, cox1, cox2, cox3, nad1, nad2, nad3, nad4, nad4L, nad5,* and *nad6*) (Li, Chen et al. 2018). Bayesian inference (BI) phylogenetic methods were used to construct phylogenetic trees using the combined gene datasets with MrBayes v3.2.6 (Ronquist et al. 2012). Bayesian posterior probabilities (BPP) were calculated to assess node support. As shown in the phylogenetic tree (Figure 1), the taxonomic status of the *Cryptococcus* sp. based on the combined mitochondrial gene dataset exhibits the closest relationship with *C. gattii* (Yadav et al. 2018).

**Figure 1.**
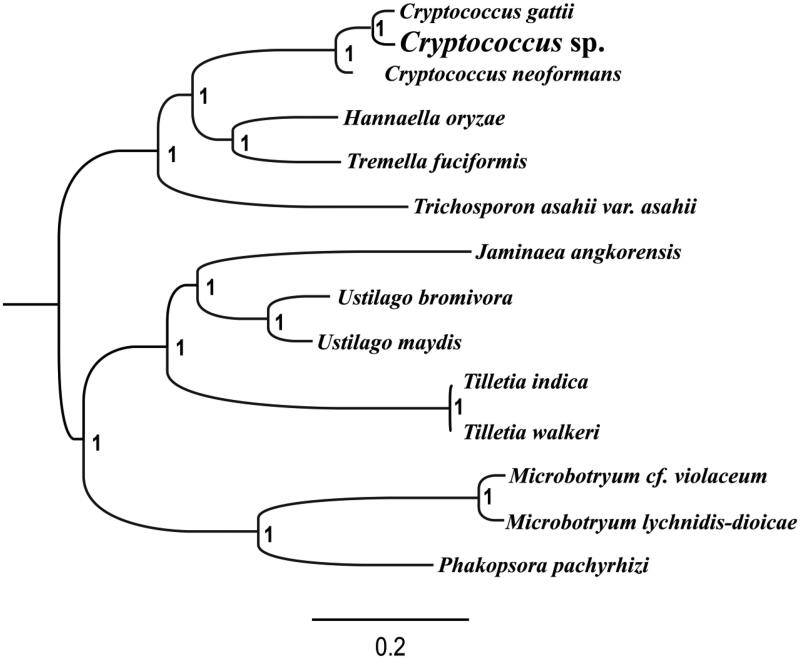
Molecular phylogenies of 14 species based on Bayesian inference analysis of the combined mitochondrial gene set (14 core protein-coding genes). Node support values are Bayesian posterior probabilities (BPP). Mitogenome accession numbers used in this phylogeny analysis: *Cryptococcus gattii* (CP025773), *Cryptococcus neoformans* (AY101381), *Hannaella oryzae* (MH732752), *Tremella fuciformis* (MF422647), *Trichosporon asahii* (JH925097), *Jaminaea angkorensis* (KC628747), *Ustilago bromivora* (LT558140), *Ustilago maydis* (DQ157700), *Tilletia indica* (DQ993184), *Tilletia walkeri* (EF536375), *Microbotryum cf. violaceum* (KC285587), *Microbotryum lychnidis-dioicae* (KC285586), *Phakopsora pachyrhizi* (GQ332420).
